# Bis[bis­(2,2′-bipyridine-κ^2^ *N*,*N*′)chloridocopper(II)] bis­(μ-2,6-pyridine­dicarboxyl­ato)-κ^4^ *O* ^2^,*N*,*O* ^6^:*O* ^6^;κ^4^ *O* ^2^:*O* ^2^,*N*,*O* ^6^-bis­[aqua­dichloridobismuthate(III)] penta­hydrate

**DOI:** 10.1107/S1600536811045259

**Published:** 2011-11-02

**Authors:** Hong-Wei Wang, Wan-Lan Liu, Yu-Quan Feng

**Affiliations:** a College of Chemistry and Pharmacy Engineering, Nanyang Normal University, Nanyang 473061, People’s Republic of China; b Department of Equipment and Lab Administration, Nanyang Normal University, Nanyang 473061, People’s Republic of China

## Abstract

In the title compound, [CuCl(C_10_H_8_N_2_)_2_]_2_[Bi_2_Cl_4_(C_7_H_3_NO_4_)_2_(H_2_O)_2_]·5H_2_O, the dianion [Bi_2_Cl_4_(C_7_H_3_NO_4_)_2_(H_2_O)_2_]^2−^ is located about an inversion center. The Cu^II^ atom of the cation is coordinated by four N atoms of the two chelating 2,2′-bypyridine ligands and one Cl^−^ ion, completing a distorted trigonal–bipyramidal coordination environment. In the anion, each Bi^III^ atom is seven-coordinate and is bonded to a tridentate pyridine-2,6-dicarboxyl­ate ligand, a water mol­ecule, two chloride ions and a bridging carboxyl­ate O atom of another carboxyl­ate ligand. The coordination geometry of Bi^III^ is distorted penta­gonal–bipyramidal with the Cl^−^ ions located in axial positions. The structure of the dianion is additionally stabilized by an intra­molecular O—H⋯O hydrogen bond between the coordinated water mol­ecule and carboxyl­ate O atom. In the crystal, O—H⋯O hydrogen bonds occur . The H atoms of the solvent water mol­ecules could not be located.

## Related literature

For examples of bis­muth(III) coordination compounds, see: Sun *et al.* (2004 ▶); Jiang *et al.* (2006 ▶); Meng & Zhang (2011 ▶).
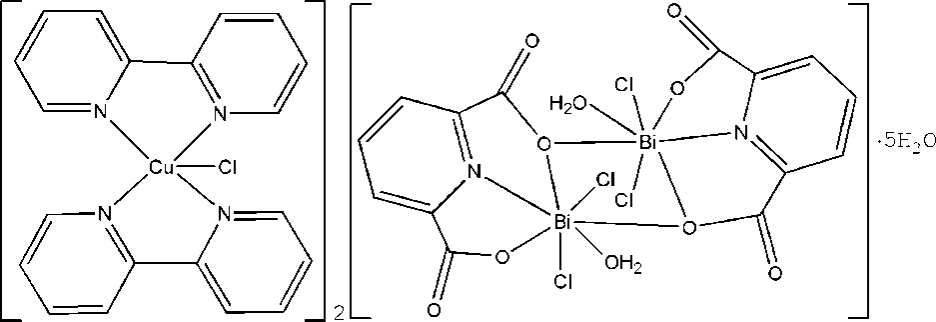


## Experimental

### 

#### Crystal data

[CuCl(C_10_H_8_N_2_)_2_]_2_[Bi_2_Cl_4_(C_7_H_3_NO_4_)_2_(H_2_O)_2_]·5H_2_O
*M* *_r_* = 1838.72
Monoclinic, 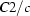

*a* = 22.880 (6) Å
*b* = 22.044 (6) Å
*c* = 13.628 (4) Å
β = 110.130 (4)°
*V* = 6454 (3) Å^3^
*Z* = 4
Mo *K*α radiation
μ = 6.41 mm^−1^
*T* = 296 K
0.18 × 0.16 × 0.15 mm

#### Data collection

Bruker APEXII CCD diffractometer
Absorption correction: multi-scan (*SADABS*; Bruker, 2008 ▶) *T*_min_ = 0.392, *T*_max_ = 0.447
16290 measured reflections
5684 independent reflections
4963 reflections with *I* > 2σ(*I*)
*R*_int_ = 0.025

#### Refinement

*R*[*F* ^2^ > 2σ(*F* ^2^)] = 0.025
*wR*(*F* ^2^) = 0.063
*S* = 1.04
5684 reflections
408 parameters
3 restraints
H atoms treated by a mixture of independent and constrained refinement
Δρ_max_ = 0.88 e Å^−3^
Δρ_min_ = −0.74 e Å^−3^

### 

Data collection: *APEX2* (Bruker, 2008 ▶); cell refinement: *SAINT* (Bruker, 2008 ▶); data reduction: *SAINT*; program(s) used to solve structure: *SHELXS97* (Sheldrick, 2008 ▶); program(s) used to refine structure: *SHELXL97* (Sheldrick, 2008 ▶); molecular graphics: *SHELXTL* (Sheldrick, 2008 ▶); software used to prepare material for publication: *SHELXTL*.

## Supplementary Material

Crystal structure: contains datablock(s) I, global. DOI: 10.1107/S1600536811045259/gk2419sup1.cif

Structure factors: contains datablock(s) I. DOI: 10.1107/S1600536811045259/gk2419Isup2.hkl

Additional supplementary materials:  crystallographic information; 3D view; checkCIF report

## Figures and Tables

**Table 1:** Selected bond lengths (Å)

Bi1—O3	2.297 (3)
Bi1—N1	2.385 (3)
Bi1—O1	2.485 (3)
Bi1—O5	2.531 (4)
Bi1—Cl2	2.6174 (15)
Bi1—Cl1	2.7479 (16)
Cl3—Cu1	2.2962 (14)
Cu1—N4	1.983 (3)
Cu1—N3	1.994 (4)
Cu1—N5	2.107 (3)
Cu1—N2	2.118 (4)

**Table 2:** Hydrogen-bond geometry (Å, °)

*D*—H⋯*A*	*D*—H	H⋯*A*	*D*⋯*A*	*D*—H⋯*A*
O5—H1⋯O3*W*	0.83 (2)	1.92 (3)	2.733 (7)	169 (11)
O5—H2⋯O2^i^	0.83 (2)	2.04 (6)	2.777 (5)	147 (10)

Symmetry code: (i) 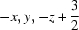.

**Fig. 1.**
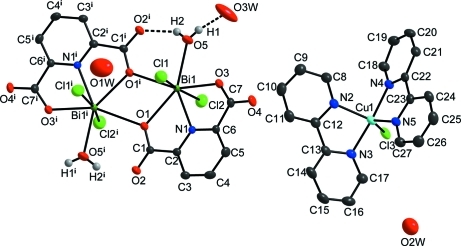
View of the title compound with displacement ellipsoids drawn at the 30% probability level (symmetry code (i): -x, y, -z+3/2).

**Fig. 2.**
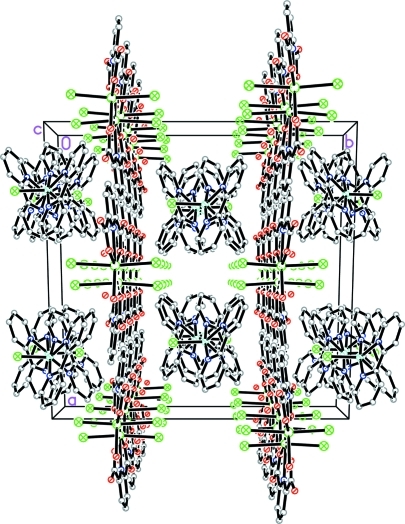
Crystal packing of the title compound along the c axis.

## supplementary crystallographic information

### Comment

Increasing attention has been paid to bismuth(III) coordination compounds in recent years due to their fascinating structural architectures and potential applications. As an expansion of bismuth(III) coordination compounds, recently, we have successfully isolated a novel compound [Cu(C_10_H_8_N_2_)_2_Cl]_2_[Bi_2_(C_7_H_3_NO_4_)_2_(H_2_O)_2_Cl_4_].5H_2_O.

The title compound consists of the coordination cations [Cu(C_10_H_8_N_2_)_2_Cl]^+^, coordination dianions [Bi_2_(C_7_H_3_NO_4_)_2_(H_2_O)_2_Cl_4_]^2-^ and solvent water molecules (Fig. 1). In coordination cation [Cu(C_10_H_8_N_2_)_2_Cl]^+^, the Cu^II^ center is coordinated by one Cl atom and four N atoms from two C_10_H_8_N_2_ ligands. In dianion [Bi_2_(C_7_H_3_NO_4_)_2_(H_2_O)_2_Cl_4_]^2-^, each Bi^III ^center is coordinated by one N atom, two Cl atoms, three O atoms and one water molecule. It is worth noting that one of the carboxylate groups acts as bridging group between two Bi centers. The structure of the dianion is additionally stabilized by intramolecular hydrogen bonds [O5—H2···O2(-*x*,*y*,-*z* + 3/2) and O(5)—H(1)···O(3 W)]. There exist the electrostatic interactions between the coordination cations and dianions, the crystal packing of the title compound along the *c* axis is shown in Fig. 2. Obviously, these electrostatic interactions and hydrogen bonds play a crucial role in the chemical stability of the title compound.

### Experimental

All chemicals were of reagent grade quality obtained from commercial sources and used without further purification. The title compound was synthesized from a mixture of Bi(NO_3_)_3_.5H_2_O (0.49 g, 1 mmol), CuCl_2_.2H_2_O(0.26 g, 1.5 mmol), 2,6-pyridinedicarboxylic acid (0.17 g, 1 mmol), 2,2'-bipyridine(0.24 g, 1.5 mmol) and H_2_O (12 g, 667 mmol) in a molar ratio of 1: 1.5: 1: 1.5: 667 by hydrothermal reaction. The mixture was stirred for half an hour, and then transferred into a Teflon-lined stainless steel autoclave (50 ml) and treated at 170 °C for 5 days. After the mixture was slowly cooled to room temperature, blue crystals were obtained. Yield: 75% (based on Bi).

### Refinement

The H atoms bonded to C were positioned geometrically and refined using a riding model, with C—H = 0.93 Å and with *U*_iso_(H) = 1.2 times *U*_eq_(C). The H atoms bonded to O5 atom were located from Fourier difference maps and refined with distance restraints of O5—H1 = 0.83 (2), O5—H2 = 0.83 (2), H1···H2 = 1.37 (2) Å. The H atoms bonded to O1W, O2W and O3W were not located in Fourier difference maps, most probably due to their disorder.

### Crystal data

**Table:**

[CuCl(C_10_H_8_N_2_)_2_]_2_[Bi_2_Cl_4_(C_7_H_3_NO_4_)_2_(H_2_O)_2_]·5H_2_O	*F*(000) = 3528
*M**_r_* = 1838.72	*D*_x_ = 1.882 Mg m^−^^3^
Monoclinic, *C*2/*c*	Mo *K*α radiation, λ = 0.71073 Å
Hall symbol: -C 2yc	Cell parameters from 8126 reflections
*a* = 22.880 (6) Å	θ = 2.4–28.1°
*b* = 22.044 (6) Å	µ = 6.41 mm^−^^1^
*c* = 13.628 (4) Å	*T* = 296 K
β = 110.130 (4)°	Block, blue
*V* = 6454 (3) Å^3^	0.18 × 0.16 × 0.15 mm
*Z* = 4	

### Data collection

**Table:**

Bruker APEXII CCD diffractometer	5684 independent reflections
Radiation source: fine-focus sealed tube	4963 reflections with *I* > 2σ(*I*)
graphite	*R*_int_ = 0.025
φ and ω scans	θ_max_ = 25.0°, θ_min_ = 1.8°
Absorption correction: multi-scan (*SADABS*; Bruker, 2008)	*h* = −27→23
*T*_min_ = 0.392, *T*_max_ = 0.447	*k* = −26→26
16290 measured reflections	*l* = −16→14

### Refinement

**Table:**

Refinement on *F*^2^	Primary atom site location: structure-invariant direct methods
Least-squares matrix: full	Secondary atom site location: difference Fourier map
*R*[*F*^2^ > 2σ(*F*^2^)] = 0.025	Hydrogen site location: inferred from neighbouring sites
*wR*(*F*^2^) = 0.063	H atoms treated by a mixture of independent and constrained refinement
*S* = 1.04	*w* = 1/[σ^2^(*F*_o_^2^) + (0.0312*P*)^2^ + 11.1454*P*] where *P* = (*F*_o_^2^ + 2*F*_c_^2^)/3
5684 reflections	(Δ/σ)_max_ = 0.002
408 parameters	Δρ_max_ = 0.88 e Å^−^^3^
3 restraints	Δρ_min_ = −0.74 e Å^−^^3^

### Fractional atomic coordinates and isotropic or equivalent isotropic displacement parameters (Å^2^)

**Table:**

	*x*	*y*	*z*	*U*_iso_*/*U*_eq_	
Bi1	0.025844 (6)	0.242343 (7)	0.610971 (11)	0.02982 (6)	
C1	0.12686 (18)	0.23152 (18)	0.8542 (3)	0.0326 (9)	
C2	0.16677 (19)	0.24402 (17)	0.7868 (3)	0.0327 (9)	
C3	0.23102 (19)	0.25200 (18)	0.8283 (4)	0.0402 (10)	
H3A	0.2521	0.2502	0.9000	0.048*	
C4	0.2625 (2)	0.2627 (2)	0.7598 (4)	0.0526 (13)	
H4A	0.3055	0.2683	0.7856	0.063*	
C5	0.2309 (2)	0.2652 (2)	0.6536 (4)	0.0457 (11)	
H5A	0.2522	0.2712	0.6073	0.055*	
C6	0.16695 (18)	0.25867 (17)	0.6173 (3)	0.0321 (9)	
C7	0.1267 (2)	0.26348 (19)	0.5032 (4)	0.0379 (10)	
C8	0.1129 (2)	0.0957 (2)	0.1789 (4)	0.0517 (12)	
H8A	0.0951	0.0875	0.1079	0.062*	
C9	0.0795 (2)	0.1308 (2)	0.2266 (4)	0.0578 (13)	
H9A	0.0403	0.1458	0.1883	0.069*	
C10	0.1061 (3)	0.1424 (2)	0.3317 (5)	0.0612 (14)	
H10A	0.0845	0.1646	0.3663	0.073*	
C11	0.1655 (2)	0.1207 (2)	0.3858 (4)	0.0525 (12)	
H11A	0.1842	0.1287	0.4567	0.063*	
C12	0.1965 (2)	0.08714 (19)	0.3336 (4)	0.0416 (10)	
C13	0.2607 (2)	0.0635 (2)	0.3840 (3)	0.0413 (10)	
C14	0.2988 (2)	0.0786 (2)	0.4839 (4)	0.0592 (13)	
H14A	0.2846	0.1048	0.5242	0.071*	
C15	0.3586 (2)	0.0543 (3)	0.5239 (4)	0.0638 (14)	
H15A	0.3848	0.0646	0.5907	0.077*	
C16	0.3783 (2)	0.0150 (2)	0.4636 (4)	0.0610 (14)	
H16A	0.4180	−0.0019	0.4889	0.073*	
C17	0.3379 (2)	0.0010 (2)	0.3640 (4)	0.0538 (12)	
H17A	0.3510	−0.0259	0.3232	0.065*	
C18	0.1123 (2)	−0.0074 (2)	−0.0053 (4)	0.0475 (11)	
H18A	0.1014	−0.0336	0.0390	0.057*	
C19	0.0703 (2)	0.0024 (2)	−0.1038 (4)	0.0530 (12)	
H19A	0.0315	−0.0163	−0.1256	0.064*	
C20	0.0872 (2)	0.0408 (2)	−0.1695 (4)	0.0538 (12)	
H20A	0.0595	0.0486	−0.2364	0.065*	
C21	0.1451 (2)	0.0674 (2)	−0.1356 (4)	0.0461 (11)	
H21A	0.1572	0.0926	−0.1799	0.055*	
C22	0.18524 (18)	0.05626 (17)	−0.0343 (3)	0.0349 (9)	
C23	0.24907 (18)	0.08145 (18)	0.0105 (3)	0.0367 (10)	
C24	0.2784 (2)	0.1136 (2)	−0.0478 (4)	0.0468 (11)	
H24A	0.2573	0.1218	−0.1181	0.056*	
C25	0.3389 (2)	0.1332 (2)	0.0002 (4)	0.0553 (13)	
H25A	0.3594	0.1540	−0.0378	0.066*	
C26	0.3683 (2)	0.1215 (2)	0.1044 (4)	0.0535 (12)	
H26A	0.4089	0.1346	0.1385	0.064*	
C27	0.3365 (2)	0.0900 (2)	0.1577 (4)	0.0488 (11)	
H27A	0.3565	0.0826	0.2286	0.059*	
Cl1	0.03209 (6)	0.36683 (7)	0.61824 (12)	0.0693 (4)	
Cl2	0.03436 (7)	0.12583 (6)	0.58147 (13)	0.0695 (4)	
Cl3	0.22828 (6)	−0.08940 (5)	0.17806 (10)	0.0560 (3)	
Cu1	0.22536 (2)	0.01472 (2)	0.17652 (4)	0.04341 (14)	
N1	0.13659 (14)	0.24776 (13)	0.6839 (3)	0.0299 (7)	
N2	0.16967 (16)	0.07324 (16)	0.2309 (3)	0.0413 (8)	
N3	0.28081 (16)	0.02474 (16)	0.3248 (3)	0.0440 (9)	
N4	0.16815 (15)	0.01917 (15)	0.0295 (3)	0.0386 (8)	
N5	0.27864 (15)	0.06980 (15)	0.1128 (3)	0.0390 (8)	
O1	0.06762 (13)	0.23135 (14)	0.8042 (2)	0.0413 (7)	
O2	0.15260 (13)	0.22296 (15)	0.9484 (2)	0.0471 (7)	
O3	0.06808 (14)	0.25476 (14)	0.4818 (2)	0.0455 (8)	
O4	0.15195 (14)	0.27517 (16)	0.4391 (2)	0.0516 (8)	
O5	−0.06953 (17)	0.2428 (2)	0.4468 (3)	0.0731 (12)	
O1W	0.0000	0.5052 (4)	0.7500	0.173 (4)	
H1	−0.073 (5)	0.257 (5)	0.389 (4)	0.208*	
H2	−0.103 (3)	0.233 (5)	0.454 (9)	0.208*	
O3W	−0.0652 (3)	0.2915 (3)	0.2650 (4)	0.151 (3)	
O2W	0.4613 (3)	−0.0920 (3)	0.3689 (4)	0.1270 (19)	

### Atomic displacement parameters (Å^2^)

**Table:**

	*U*^11^	*U*^22^	*U*^33^	*U*^12^	*U*^13^	*U*^23^
Bi1	0.02219 (9)	0.04836 (10)	0.01923 (9)	0.00043 (6)	0.00754 (6)	−0.00092 (6)
C1	0.028 (2)	0.044 (2)	0.025 (2)	0.0032 (17)	0.0069 (17)	−0.0018 (17)
C2	0.029 (2)	0.038 (2)	0.029 (2)	0.0015 (15)	0.0080 (18)	−0.0032 (17)
C3	0.027 (2)	0.057 (3)	0.032 (3)	−0.0022 (17)	0.0046 (18)	−0.0015 (19)
C4	0.022 (2)	0.083 (3)	0.047 (3)	−0.005 (2)	0.004 (2)	0.007 (2)
C5	0.034 (2)	0.064 (3)	0.045 (3)	−0.005 (2)	0.021 (2)	0.002 (2)
C6	0.028 (2)	0.042 (2)	0.028 (2)	−0.0020 (16)	0.0118 (18)	−0.0032 (17)
C7	0.037 (2)	0.049 (2)	0.032 (2)	−0.0020 (18)	0.018 (2)	−0.0033 (19)
C8	0.055 (3)	0.054 (3)	0.044 (3)	0.006 (2)	0.014 (2)	0.003 (2)
C9	0.053 (3)	0.059 (3)	0.063 (4)	0.001 (2)	0.022 (3)	0.002 (3)
C10	0.069 (4)	0.059 (3)	0.067 (4)	0.000 (3)	0.039 (3)	−0.008 (3)
C11	0.064 (3)	0.051 (3)	0.046 (3)	−0.012 (2)	0.023 (2)	−0.009 (2)
C12	0.047 (3)	0.040 (2)	0.039 (3)	−0.0089 (19)	0.017 (2)	0.002 (2)
C13	0.049 (3)	0.046 (2)	0.030 (2)	−0.012 (2)	0.015 (2)	0.0052 (19)
C14	0.063 (3)	0.079 (4)	0.035 (3)	−0.017 (3)	0.016 (2)	−0.009 (3)
C15	0.059 (3)	0.089 (4)	0.035 (3)	−0.017 (3)	0.006 (3)	−0.002 (3)
C16	0.048 (3)	0.073 (4)	0.051 (3)	−0.005 (2)	0.002 (2)	0.014 (3)
C17	0.053 (3)	0.060 (3)	0.046 (3)	0.004 (2)	0.013 (2)	0.008 (2)
C18	0.043 (3)	0.055 (3)	0.041 (3)	−0.008 (2)	0.011 (2)	0.005 (2)
C19	0.038 (2)	0.062 (3)	0.052 (3)	−0.003 (2)	0.007 (2)	−0.003 (2)
C20	0.046 (3)	0.067 (3)	0.037 (3)	0.007 (2)	−0.001 (2)	−0.001 (2)
C21	0.052 (3)	0.049 (3)	0.037 (3)	0.005 (2)	0.016 (2)	0.006 (2)
C22	0.040 (2)	0.035 (2)	0.032 (2)	0.0012 (17)	0.0138 (18)	−0.0058 (18)
C23	0.045 (3)	0.033 (2)	0.036 (3)	0.0007 (17)	0.020 (2)	−0.0029 (18)
C24	0.060 (3)	0.046 (3)	0.038 (3)	−0.008 (2)	0.021 (2)	−0.003 (2)
C25	0.067 (3)	0.048 (3)	0.061 (4)	−0.019 (2)	0.036 (3)	−0.009 (2)
C26	0.049 (3)	0.052 (3)	0.060 (4)	−0.015 (2)	0.019 (3)	−0.009 (2)
C27	0.047 (3)	0.054 (3)	0.041 (3)	−0.005 (2)	0.009 (2)	−0.003 (2)
Cl1	0.0662 (9)	0.0669 (8)	0.0728 (10)	−0.0064 (6)	0.0214 (7)	−0.0097 (7)
Cl2	0.0781 (9)	0.0537 (7)	0.0808 (10)	0.0080 (6)	0.0326 (8)	0.0003 (7)
Cl3	0.0835 (9)	0.0496 (6)	0.0356 (6)	0.0040 (6)	0.0213 (6)	0.0012 (5)
Cu1	0.0458 (3)	0.0514 (3)	0.0292 (3)	−0.0035 (2)	0.0081 (2)	0.0028 (2)
N1	0.0232 (16)	0.0408 (19)	0.0261 (19)	0.0002 (12)	0.0092 (14)	−0.0019 (14)
N2	0.045 (2)	0.046 (2)	0.034 (2)	−0.0004 (16)	0.0153 (17)	0.0033 (16)
N3	0.044 (2)	0.050 (2)	0.033 (2)	−0.0016 (17)	0.0084 (17)	0.0047 (17)
N4	0.0390 (19)	0.044 (2)	0.031 (2)	−0.0048 (15)	0.0089 (15)	−0.0021 (16)
N5	0.0378 (19)	0.046 (2)	0.031 (2)	−0.0052 (15)	0.0085 (16)	−0.0026 (16)
O1	0.0284 (15)	0.070 (2)	0.0261 (16)	0.0004 (13)	0.0101 (12)	0.0002 (14)
O2	0.0369 (16)	0.077 (2)	0.0258 (17)	0.0039 (15)	0.0083 (13)	0.0036 (15)
O3	0.0317 (16)	0.080 (2)	0.0268 (17)	−0.0045 (13)	0.0126 (13)	−0.0020 (14)
O4	0.0481 (18)	0.082 (2)	0.0329 (18)	−0.0098 (16)	0.0247 (15)	−0.0006 (17)
O5	0.036 (2)	0.143 (4)	0.032 (2)	0.0002 (19)	0.0021 (16)	0.001 (2)
O1W	0.187 (9)	0.111 (6)	0.191 (11)	0.000	0.026 (8)	0.000
O3W	0.221 (6)	0.136 (5)	0.053 (3)	−0.077 (5)	−0.009 (4)	0.012 (3)
O2W	0.149 (5)	0.119 (4)	0.110 (5)	0.006 (4)	0.040 (4)	−0.011 (3)

### Geometric parameters (Å, °)

**Table:**

Bi1—O3	2.297 (3)	C14—H14A	0.9300
Bi1—N1	2.385 (3)	C15—C16	1.373 (8)
Bi1—O1	2.485 (3)	C15—H15A	0.9300
Bi1—O5	2.531 (4)	C16—C17	1.390 (7)
Bi1—Cl2	2.6174 (15)	C16—H16A	0.9300
Bi1—Cl1	2.7479 (16)	C17—N3	1.336 (5)
C1—O2	1.229 (5)	C17—H17A	0.9300
C1—O1	1.290 (5)	C18—N4	1.336 (5)
C1—C2	1.527 (6)	C18—C19	1.372 (6)
C2—N1	1.335 (5)	C18—H18A	0.9300
C2—C3	1.393 (6)	C19—C20	1.380 (7)
C3—C4	1.382 (7)	C19—H19A	0.9300
C3—H3A	0.9300	C20—C21	1.375 (6)
C4—C5	1.379 (7)	C20—H20A	0.9300
C4—H4A	0.9300	C21—C22	1.392 (6)
C5—C6	1.382 (6)	C21—H21A	0.9300
C5—H5A	0.9300	C22—N4	1.347 (5)
C6—N1	1.341 (5)	C22—C23	1.483 (5)
C6—C7	1.515 (6)	C23—N5	1.349 (5)
C7—O4	1.229 (5)	C23—C24	1.396 (6)
C7—O3	1.285 (5)	C24—C25	1.381 (6)
C8—N2	1.341 (6)	C24—H24A	0.9300
C8—C9	1.395 (7)	C25—C26	1.371 (7)
C8—H8A	0.9300	C25—H25A	0.9300
C9—C10	1.374 (7)	C26—C27	1.381 (6)
C9—H9A	0.9300	C26—H26A	0.9300
C10—C11	1.390 (7)	C27—N5	1.329 (5)
C10—H10A	0.9300	C27—H27A	0.9300
C11—C12	1.380 (6)	Cl3—Cu1	2.2962 (14)
C11—H11A	0.9300	Cu1—N4	1.983 (3)
C12—N2	1.355 (6)	Cu1—N3	1.994 (4)
C12—C13	1.485 (6)	Cu1—N5	2.107 (3)
C13—N3	1.360 (6)	Cu1—N2	2.118 (4)
C13—C14	1.380 (6)	O5—H1	0.83 (2)
C14—C15	1.393 (7)	O5—H2	0.83 (2)
			
O3—Bi1—N1	69.30 (11)	N3—C17—C16	122.3 (5)
O3—Bi1—O1	135.54 (10)	N3—C17—H17A	118.9
N1—Bi1—O1	66.26 (10)	C16—C17—H17A	118.9
O3—Bi1—O5	77.55 (13)	N4—C18—C19	122.7 (4)
N1—Bi1—O5	146.83 (13)	N4—C18—H18A	118.6
O1—Bi1—O5	146.85 (12)	C19—C18—H18A	118.6
O3—Bi1—Cl2	85.76 (8)	C18—C19—C20	118.2 (4)
N1—Bi1—Cl2	89.34 (8)	C18—C19—H19A	120.9
O1—Bi1—Cl2	92.60 (8)	C20—C19—H19A	120.9
O5—Bi1—Cl2	87.41 (12)	C19—C20—C21	119.8 (4)
O3—Bi1—Cl1	83.01 (8)	C19—C20—H20A	120.1
N1—Bi1—Cl1	84.28 (8)	C21—C20—H20A	120.1
O1—Bi1—Cl1	93.61 (8)	C20—C21—C22	119.2 (4)
O5—Bi1—Cl1	92.67 (12)	C20—C21—H21A	120.4
Cl2—Bi1—Cl1	168.47 (5)	C22—C21—H21A	120.4
O2—C1—O1	126.1 (4)	N4—C22—C21	120.6 (4)
O2—C1—C2	119.0 (3)	N4—C22—C23	115.2 (4)
O1—C1—C2	114.9 (4)	C21—C22—C23	124.2 (4)
N1—C2—C3	120.7 (4)	N5—C23—C24	121.1 (4)
N1—C2—C1	116.3 (3)	N5—C23—C22	115.3 (3)
C3—C2—C1	123.0 (4)	C24—C23—C22	123.6 (4)
C4—C3—C2	118.1 (4)	C25—C24—C23	119.3 (4)
C4—C3—H3A	121.0	C25—C24—H24A	120.4
C2—C3—H3A	121.0	C23—C24—H24A	120.4
C5—C4—C3	120.5 (4)	C26—C25—C24	119.2 (4)
C5—C4—H4A	119.7	C26—C25—H25A	120.4
C3—C4—H4A	119.7	C24—C25—H25A	120.4
C6—C5—C4	118.7 (4)	C25—C26—C27	118.6 (4)
C6—C5—H5A	120.6	C25—C26—H26A	120.7
C4—C5—H5A	120.6	C27—C26—H26A	120.7
N1—C6—C5	120.5 (4)	N5—C27—C26	123.3 (5)
N1—C6—C7	115.7 (3)	N5—C27—H27A	118.4
C5—C6—C7	123.8 (4)	C26—C27—H27A	118.4
O4—C7—O3	125.5 (4)	N4—Cu1—N3	170.69 (14)
O4—C7—C6	118.4 (4)	N4—Cu1—N5	80.20 (13)
O3—C7—C6	116.1 (4)	N3—Cu1—N5	95.56 (14)
N2—C8—C9	123.0 (5)	N4—Cu1—N2	93.21 (14)
N2—C8—H8A	118.5	N3—Cu1—N2	80.07 (15)
C9—C8—H8A	118.5	N5—Cu1—N2	107.23 (14)
C10—C9—C8	118.2 (5)	N4—Cu1—Cl3	93.77 (10)
C10—C9—H9A	120.9	N3—Cu1—Cl3	95.46 (11)
C8—C9—H9A	120.9	N5—Cu1—Cl3	124.09 (10)
C9—C10—C11	119.2 (5)	N2—Cu1—Cl3	128.65 (10)
C9—C10—H10A	120.4	C2—N1—C6	121.4 (3)
C11—C10—H10A	120.4	C2—N1—Bi1	121.7 (3)
C12—C11—C10	119.7 (5)	C6—N1—Bi1	116.8 (3)
C12—C11—H11A	120.2	C8—N2—C12	118.3 (4)
C10—C11—H11A	120.2	C8—N2—Cu1	129.0 (3)
N2—C12—C11	121.4 (4)	C12—N2—Cu1	112.6 (3)
N2—C12—C13	115.1 (4)	C17—N3—C13	119.5 (4)
C11—C12—C13	123.5 (4)	C17—N3—Cu1	123.7 (3)
N3—C13—C14	120.6 (4)	C13—N3—Cu1	116.4 (3)
N3—C13—C12	115.4 (4)	C18—N4—C22	119.4 (4)
C14—C13—C12	124.0 (4)	C18—N4—Cu1	123.7 (3)
C13—C14—C15	119.7 (5)	C22—N4—Cu1	116.5 (3)
C13—C14—H14A	120.1	C27—N5—C23	118.5 (4)
C15—C14—H14A	120.1	C27—N5—Cu1	129.0 (3)
C16—C15—C14	119.2 (5)	C23—N5—Cu1	112.3 (3)
C16—C15—H15A	120.4	C1—O1—Bi1	120.7 (2)
C14—C15—H15A	120.4	C7—O3—Bi1	121.7 (3)
C15—C16—C17	118.6 (5)	Bi1—O5—H1	128 (8)
C15—C16—H16A	120.7	Bi1—O5—H2	117 (8)
C17—C16—H16A	120.7	H1—O5—H2	114 (5)

### Hydrogen-bond geometry (Å, °)

**Table:**

*D*—H···*A*	*D*—H	H···*A*	*D*···*A*	*D*—H···*A*
O5—H1···O3W	0.83 (2)	1.92 (3)	2.733 (7)	169 (11)
O5—H2···O2^i^	0.83 (2)	2.04 (6)	2.777 (5)	147 (10)

Symmetry codes: (i) −*x*, *y*, −*z*+3/2.

## References

[bb1] Bruker (2008). *APEX2*, *SAINT* and *SADABS* Bruker AXS Inc., Madison, Wisconsin, USA.

[bb2] Jiang, Q. Y., Shen, J. & Zhong, G. Q. (2006). *Chin. J. Prog. Chem.* **18**, 1634–1645.

[bb3] Meng, Z.-H. & Zhang, S.-S. (2011). *Acta Cryst.* E**67**, m1402–m1403. 10.1107/S160053681103769X PMC3201447 22065819

[bb4] Sheldrick, G. M. (2008). *Acta Cryst.* A**64**, 112–122. 10.1107/S0108767307043930 18156677

[bb5] Sun, H., Zhang, L. & Szeto, K. Y. (2004). *Met. Ions Biol. Syst.* **41**, 333–378. 15206122

